# Efects of the preoperative use of artificial tears combined with recombinant bovine basic fibroblast growth factor on cataract patients complicated with dry eyes

**DOI:** 10.5935/0004-2749.2021-0539

**Published:** 2022-09-06

**Authors:** Wei Xiao, Pengcheng Huang

**Affiliations:** 1 Department of Cataract and Glaucoma, Hankou Aier Eye Hospital, Wuhan 430000, Hubei Province, China; 2 School of Clinical Medicine, Weifang Medical University, Weifang 261061, Shandong Province, China; 3 Department of Cataract and Glaucoma, The Eyegood Eye Hospital of Wuhan, Wuhan 430019, Hubei Province, China

**Keywords:** Artificial tear, Basic fibroblast growth factor, Ocular surface, Cataract, Dry eyes, Lágrima artificial, Fator básico de crescimento de fibroblasto, Superfície ocular, Catarata, Olho seco

## Abstract

**Purpose:**

To assess the effects of the preoperative application of artificial tears combined with recombinant bovine basic fibroblast growth factor on the ocular surface function and inflammatory factor levels after operation in cataract patients complicated with dry eyes.

**Methods:**

A total of 118 cataract patients (118 eyes) complicated with dry eyes treated from February 2019 to February2020 were assigned to control and observation groups (n=59 eyes/group) using a random number table. One week before the operation, the control group was administered 0.1% sodium hyaluronate eye drops (artificial tears), based on which the observation group received Beifushu eye drops (recombinant bovine basic fibroblast growth factor), both 6 times daily for 1 week. A comparison was made between the scores of clinical symptoms and the indices of ocular surface function, inflammatory factors in tears, and oxidative stress indices before and after the operation. The ocular surface function was evaluated by an ocular surface disease index questionnaire, tear film breakup-time assay, Schirmer’s I test, and corneal fluorescein stain test. The inflammatory factors in tears were measured.

**Results:**

No significant differences were noted in the general data and clinical symptom score, ocular surface disease index, tear film breakup-time, Schirmer’s I test score, fluorescein stain score, interleukin-6, tumor necrosis factor-alpha, malondialdehyde, superoxide dismutase, lipid peroxide, and total antioxidant capacity before treatment between the 2 groups (p>0.05). After treatment, the clinical symptom score, ocular surface disease index, fluorescein stain score, tumor necrosis factor-alpha, interleukin-6, malondial-dehyde and lipid peroxide declined significantly, and tear film breakup-time, Schirmer’s I test score, superoxide dismutase, and total antioxidant capacity increased in both the groups. The improvements in the clinical symptom score as well as in the indices of ocular surface function, inflammatory factors, and oxidative stress were more prominent in the observation group than in the control group (p<0.05).

**Conclusions:**

Artificial tears combined with recombinant bovine basic fibroblast growth factor before operation. significantly improved the ocular surface function, reduced inflammatory factors in tears, and alleviated dry eye symptoms after operation in cataract patients.

## Abstract (pt)

**Objetivo:**

Avaliar os efeitos da aplicação pré-operatória de lágrimas artificiais combinadas com o fator de crescimento de fibroblastos básicos bovinos recombinantes na função da superfície ocular e níveis de fator inflamatório após cirurgia em pacientes com catarata complicada com olhos secos.

**Métodos:**

Um total de 118 pacientes com catarata complicada com olhos secos (118 olhos), tratados entre fevereiro de 2019 e fevereiro de 2020, foram divididos em grupos de controle e de observação (n=59, 59 olhos) usando uma tabela de números aleatórios. Uma semana antes da cirurgia, o grupo controle recebeu colírio de hialuronato de sódio a 0,1% (lágrimas artificiais), enquanto o grupo de observação recebeu colírio Beifushu (fator de crescimento de fibroblastos básicos bovinos recombinantes), ambos, seis vezes ao dia, por uma semana. Antes do tratamento e um mês após a cirurgia, os escores de sintomas clínicos, índices de função da superfície ocular, níveis de fatores inflamatórios nas lágrimas e índices de estresse oxidativo foram comparados. A função da superfície ocular foi avaliada pelo questionário do índice de doença da superfície ocular, ensaio de tempo de ruptura do filme lacrimal, teste I de Schirmer e teste de coloração por fluoresceína da córnea. Os níveis de fatores inflamatórios nas lágrimas foram medidos.

**Resultados:**

Não houve diferenças significativas nos dados gerais e no escore de sintomas clínicos, índice de doença da superfície ocular, tempo de ruptura do filme lacrimal, escore do teste I de Schirmer, pontuação do teste de coloração por fluoresceína da córnea, interleucina-6, fator de necrose tumoral alfa, malondialdeído, superóxido dismutase, peróxido lipídico e capacidade antioxidante total antes do tratamento entre os dois grupos (p>0,05). Após o tratamento, o escore de sintomas clínicos, índice de doença da superfície ocular, escore do teste de coloração por fluoresceína da córnea, fator de necrose tumoral alfa, interleucina-6, malondialdeído e peróxido lipídico diminuíram significativamente, e o tempo de ruptura do filme lacrimal, escore do teste I de Schirmer, superóxido dismutase e a capacidade antioxidante total aumentou em ambos os grupos. As melhorias no escore de sintomas clínicos, bem como os índices de função da superfície ocular, fatores inflamatórios e estresse oxidativo foram mais proeminentes no grupo de observação do que no grupo controle (p<0,05).

**Conclusões:**

Lágrimas artificiais combinadas com fator de crescimento de fibroblastos básicos recombinantes antes da cirurgia melhoram notavelmente a função da superfície ocular, diminuem os níveis de fatores inflamatórios nas lágrimas e aliviam os sintomas de olho seco após a cirurgia em pacientes com catarata complicada com olhos secos.

## INTRODUCTION

A cataract is an ophthalmic disease that frequently occurs in the elderly, wherein the degeneration of the lens is caused by metabolic disorders owing to multiple factors which finally result in lens opacity^(1)^. It usually occurs in the elderly, and its morbidity rate is increasing with population aging, reaching 65.78% among men aged 85-89 years^(2)^. The present therapeutic method for cataracts offers advantages such as less trauma, rapid recovery, and prominent efficacy^(3)^. However, the corneal epithelium is damaged, and massive inflammatory factors are synthesized and secreted by the ocular surface epithelial cells in the process of phacoemulsification^(4)^. Dry eyes following cataract surgery are a frequent ocular surface inflammatory disease in clinical ophthalmology, with ocular surface symptoms and the imbalance of tear film homeostasis as the major manifestations. It can result in vision disorders, affect the daily lives of patients, and even cause blindness in severe cases if not treated on time^(5)^. A past study demonstrated that artificial tears can efficiently relieve dry eye symptoms in patients after cataract surgery and restore the stability of the tear film^(6)^. Basic fibroblast growth factor (bFGF), a physiological component in the normal corneal tissues, can promote repair and regeneration^(7)^. It has been shown that artificial tears combined with recombinant bFGF (rbFGF) can improve the ocular surface function and enhance the therapeutic effect on dry eyes after cataract surgery^(8)^. However, the dry eye symptoms of patients complicated with dry eyes before cataract surgery is aggravated after surgery, thereby affecting the therapeutic effect on patients. Nowadays, preoperative intervention in dry eye symptoms required for cataract patients complicated with dry eyes is rarely reported. Therefore, the influences of preoperative application of artificial tears combined with rbFGF on the ocular surface function and the levels of inflammatory factors after operation in cataract patients complicated with dry eyes were explored in this study so as to provide a reference for clinical treatment of cataract complicated with dry eyes.

## METHODS

### General data

A total of 118 cataract patients (118 eyes) complicated with dry eyes were admitted to and treated at our hospital during February 2019-2020. The patients were then assigned into control group (n=59, 59 eyes) and observation group (n=59, 59 eyes) using a random number table. The control group patients (32 men [32 eyes], 27 women [27 eyes]; age 50-76 years; average age 62.05 ± 6.32 years) were treated with artificial tears. The course of the disease was 1-5 years (average: 2.68 ± 0.79 years). The observation group patients (35 men [35 eyes], 24 women [24 eyes]; age: 52-75 years; average age: 62.46 ± 6.58 years) were administered artificial tears combined with rbFGF. The course of disease in this group was 1-5 years (average: 2.72 ± 0.82). This study was reviewed and approved by the Medical Ethics Committee of the hospital, and all patients and their families provided their signed informed consent.

### Inclusion and exclusion criteria

The inclusion criteria were set as follows: 1) patients meeting the diagnostic criteria for age-related cataracts^(9)^ (lens opacity and best-corrected visual acuity <20/40), 2) those meeting the diagnostic criteria for dry eyes^(10)^ and diagnosed with dry eyes before the operation, 3) those without other complications after the operation, 4) those who willingly completed the questionnaire survey and related examinations, and 5) those with complete clinical data.

The following exclusion criteria were adopted: 1) patients with a history of eye surgery or trauma; 2) those with diabetes mellitus, Sjögren’s syndrome, or other systemic diseases; 3) those with ocular fundus hemorrhage, keratitis, or other ophthalmic diseases; 4) those who used drugs affecting the tear film stability for a long time; or 5) those with hematologic disease or severe infection.

### Collection of general data

The general data, including the age, gender, and course of the disease, of the patients were collected through electronic medical records.

### Operative methods

Preoperative intervention: At 1 week before the operation, the patients in the control group were provided 0.1% sodium hyaluronate eye drops (artificial tears) 6 times a day. On this basis, the patients in the observation group additionally received the Beifushu eye drops (rbFGF) 6 times a day.

The participants were operated on by the same surgeon. After using topical anesthesia in the operative region, the transparent cornea was cut open, and a viscoelastic agent was injected into the anterior chamber. Later, the cortex, anterior capsule, and the nucleus of the lens were isolated *via* continuous circular capsulorhexis and water separation; phacoemulsification was performed for the lens nucleus, and an artificial lens was implanted.

As for the postoperative treatments, the patients in both groups were smeared with tobramycin and dexamethasone eye ointment in the conjunctival sac after the operation. In addition, levofloxacin eye drops, 0.1% sodium hyaluronate eye drops, tobramycin and dexamethasone eye drops, and deproteinized calf serum-containing eye gel were routinely applied 4 times a day; the doses were decreased every week, and the drugs were withdrawn 1 month later.

### Observation indices

The clinical symptoms of the patients before and after treatment were scored according to the *Guiding Principles for Clinical Study of New Chinese Medicines*^(11)^. Specifically, the symptom of eye dryness was scored 0, 2, 4, and 6 points, and foreign body sensation, asthenopia, photophobia, and red-eye were scored 0, 1, 2, and 3 points, respectively. The sum of the scores of all symptoms was recorded as the total score of clinical symptoms, and a higher score indicated severer symptoms.

The ocular surface function of the patients before and after treatment was evaluated through the ocular surface disease index (OSDI) questionnaire, tear film breakup-time (BUT) assay, Schirmer’s I test (SIt), and corneal fluorescein stain test (FL). The OSDI questionnaire was composed of 12 items and scored in the range of 0-100 points in total. The higher the score, the more serious were the ocular surface symptoms. As for the BUT, a fluo rescein sodium ophthalmic test strip wetted by normal saline was placed under the conjunctival sac of the lower eyelid, the tear film was observed using a cobalt-blue slit lamp, and the duration from the fourth blink to the occurrence of black lines or black spots was recorded by a stopwatch (regarded as the BUT). In the SIt, the upper end of the Schirmer tear strip was turned over by 5 mm and then placed into the conjunctival sac at mid-lateral 1/3^rd^ of the lower eyelid. After closing the eyes gently for 5 min, the strip was removed to measure the wetting length away from the turnover, and the length <10 mm/5 min indicated abnormal tear secretion volume. During the FL, the fluorescein sodium solution was dripped into the conjunctival sac of the lower eyelid, and the patients were instructed to blink naturally 4 times, followed by observation under the cobalt-blue slit lamp. The ocular staining score (OSS) method was utilized. The ocular surface of each eye was divided into 3 parts (nasal conjunctiva, cornea, and temporal conjunctiva). The conjunctiva was scored according to the number of stained dots in the palpebral fissure area, as follows: 0 point: 0-9 stained dots; 1 point: 10-32 stained dots; 2 points: 33-100 stained dots; 3 points: >100 stained dots. The cornea was scored according to the number, shape, and distribution of the stained dots, as follows: 0 points: without stained dots; 1 point: 1-5 stained dots; 2 points: 6-30 stained dots; 3 points: >30 stained dots. If the stained dots were fused or located in the pupil area or if there was filiform keratitis, 1 additional point was added to the above score. OSS of a single eye was the sum of the scores of nasal conjunctiva, cornea, and temporal conjunctiva, with a maximum of 12 points^(12)^. OSDI was first evaluated, followed by BUT, Sit, and FL sequentially. The latter three tests were performed at an interval of 2 h.

The levels of inflammatory factors in the tears and the oxidative stress indices were detected. Specifically, the tears were collected with a capillary tube before and after treatment. The total volume was kept to 10 µL. Next, the levels of interleukin-6 (IL-6), tumor necrosis factor-α (TNF-α), malondialdehyde (MDA), lipid peroxide (LPO), and superoxide dismutase (SOD) were determined through enzyme-linked immunosorbent assay^(13)^. Finally, the total antioxidant capacity (TAC) was examined by 2,2’-azinobis-(3-ethylbenzthiazo line-6-sulphonate) colorimetry.

### Statistical analysis

SPSS 19.0 software was employed for statistical analysis, and the GraphPad Prism 5.0 software was used for plotting. The numerical data were represented as a percentage, and a Chi-square test was performed for comparison between the groups. The measurement data were expressed as mean ± standard deviation, an independent *t*-test was adopted for intergroup comparison, and paired *t*-test was used for comparison within the group. P<0.05 was considered to indicate statistical significance.

## RESULTS

### General data

There were no significant differences in the age, gender, and course of disease between the 2 groups (p>0.05) (Table 1).

**Table 1:** General data

Group	Control group (n=59)	Observation group (n=59)	*t*/χ^2^	p-value
Age (year)	62.05 ± 6.32	62.46 ± 6.58	0.345	0.731
Male/female (n)	32/27	35/24	0.311	0.577
Course of disease (year)	2.68 ± 0.79	2.72 ± 0.82	0.270	0.788

### Clinical symptom scores before and after treatment

The difference in the clinical symptom score was not significant before treatment between the two groups of patients (p>0.05). The clinical symptom score decreased significantly in both groups after treatment compared with that before treatment, and it was notably lower in the observation group than in the control group (p<0.05) (Table 2).

**Table 2:** Clinical symptom score before and after treatment

Group	Control group (n=59)	Observation group (n=59)	t	p-value
Before treatment	10.62 ± 2.12	10.57 ± 2.08	0.129	0.897
After treatment	5.02 ± 0.22	1.73 ± 0.35	61.130	<0.001
*t*	20.181	32.192	-	-
p	<0.001	<0.001	-	-

### Indices of ocular surface function before and after treatment

The OSDI, BUT, SIt score, and FL score had no significant differences before treatment between the two groups of patients (p>0.05). After treatment, however, the BUT and SIt score increased significantly, while the OSDI and FL score declined significantly in the two groups in contrast with those before treatment. In addition, the observation group exhibited significantly longer BUT, higher SIt score, and lower OSDI and FL scores than the control group after treatment (p<0.05) (Table 3).

**Table 3:** Indices of the ocular surface function before and after treatment

Group	Control group (n=59)		Observation group (n=59)	
	Before treatment	After treatment	Before treatment	After treatment
OSDI (point)	39.62 ± 8.45	32.53 ± 6.62*	38.93 ± 9.06	24.56 ± 6.05*^#^
BUT (s)	3.77 ± 0.62	5.72 ± 1.08*	3.73 ± 0.68	9.82 ± 1.31*^#^
SIt (mm/5 min)	5.28 ± 1.05	7.81 ± 1.32*	5.25 ± 1.02	11.18 ± 2.18*^#^
FL score (point)	4.93 ± 0.73	2.24 ± 0.42*	4.96 ± 0.68	0.95 ± 0.21*^#^

*p<0.05 *vs.* before treatment within the group, ^#^p<0.05 *vs.* control group after treatment.

### Levels of infammatory factors in tears before and after treatment

Between the two groups of patients, there were no significant differences in the IL-6 and TNF-* levels before treatment (p>0.05). The TNF-* and IL-6 levels were significantly lower in the observation group compared with that in the control group (p * 0.05) after treatment than before treatment (Table 4).

**Table 4:** Levels of infammatory factors in tears before and after treatment

Groups	IL-6 (pg/mL)		TNF-α (pg/mL)	
	Before treatment	After treatment	Before treatment	After treatment
Control group (n=59)	1432.72 ± 112.43	1385.16 ± 75.31*	259.53 ± 19.73	247.62 ± 22.85*
Observation group (n=59)	1425.64 ± 108.46	1148.65 ± 71.37*	265.32 ± 18.69	187.59 ± 23.47*
*t*	0.348	17.509	1.636	39.606
p	0.728	<0.001	0.104	<0.001

*p<0.05 vs. before treatment within the group.

### Oxidative stress indices in tears before and after treatment

No significant differences in MDA, SOD, LPO, and TAC were detected between the two groups of patients before treatment (p>0.05). After treatment, the MDA and LPO reduced significantly, and the SOD and TAC were raised significantly in the two groups when compared with those before treatment. Furthermore, the changes in the above indices were significantly more prominent in the observation group than in the control groups after treatment (p<0.05) (Table 5).

**Table 5:** Oxidative stress indices in tears before and after treatment

Group	Control group (n=59)		Observation group (n=59)	
	Before treatment	After treatment	Before treatment	After treatment
MDA (U/L)	7.08 ± 0.96	3.28 ± 0.15*	7.12 ± 0.98	2.32 ± 0.18*^#^
SOD (mmol/L)	0.08 ± 0.03	0.12 ± 0.04*	0.08 ± 0.02	0.17 ± 0.05*^#^
LPO (µmol/L)	2.17 ± 0.45	1.59 ± 0.32*	2.19 ± 0.47	1.11 ± 0.28*^#^
TAC (kU/L)	7.58 ± 0.83	10.28 ± 1.86*	7.55 ± 0.96	14.62 ± 2.13*^#^

*p<0.05 *vs.* before treatment within the group, ^#^p<0.05 *vs.* control group after treatment.

## DISCUSSION

As a common ocular surface inflammatory disease in clinical ophthalmology, dry eyes refer to the loss of tear film homeostasis and ocular surface disease caused by multiple factors such as ocular surface inflammation, tear hypertonicity, nerve sensory abnormality, and tear film instability^(14)^. The clinical symptoms of dry eyes include foreign body sensation, photophobia, asthenopia, eye dryness, burning sensation, itching, and hyperemia ^(15)^. The prevalence rate of dry eyes ranges from around 2.1% to 35%^(16)^. Cataract surgery is an important cause of dry eyes, and the morbidity rate of dry eyes is as high as 70% 1 week after cataract surgery^(17)^. Dry eyes can be recovered in the majority of patients, but it is slowly restored or not restored in 20% of patients^(18)^. Currently, dry eyes after cataract surgery are mainly treated by supplementing tears in the clinic, which can alleviate eye dryness and burning sensation of patients, thereby ameliorating patients’ visual quality^(19)^. Clinically, many cataract patients develop dry eyes before surgery, and the symptoms of dry eyes worsen after surgery^(20)^, thereby affecting the postoperative recovery and therapeutic efficacy. Hence, it is necessary to conduct an intervention in preoperative dry eye symptoms in cataract patients complicated with dry eyes to mitigate dry eye symptoms after operation. Nevertheless, there are a few reports on the intervention in dry eye symptoms in cataract patients complicated with dry eyes before the operation at the present^(21)^. Therefore, the influences of preoperative application of artificial tears combined with rbFGF on the ocular surface function and the levels of inflammatory factors after operation in cataract patients complicated with dry eyes were explored in the study.

Artificial tears, a type of protective clear hydrogel, can maintain the moisture of the eyes, promote the repair of cells, tear film, and improve the tear film stability, thereby relieving the ocular surface symptoms, ameliorating the visual quality of patients, and providing in a preferable short-term therapeutic effect^(22)^. RbFGF can facilitate repair and regeneration as well as promote corneal epithelial repair through various signaling pathways^(23)^. The overall objective of dry eye treatment is to restore the normal structure, and function of the ocular surface, and inhibit inflammatory response on the ocular surface^(24)^. You et al.^(25)^ explored the therapeutic effect of artificial tears on patients with dry eyes after cataract surgery. They found that artificial tears reduced the subjective symptom score of dry eyes and FL score remarkably, obviously increased the BUT and SIt score, and notably improved the ocular surface function of patients. In this study, the observation group showed significantly longer BUT high SIt scores, and low OSDI and FL scores than the control group after treatment, suggesting that the combination of artificial tears with rbFGF can relieve early dry eyes after surgery. Yoon et al.^(26)^ evaluated the application effect of rbFGF on dry eyes patients after cataract surgery. It was discovered that BUT was distinctly prolonged, the SIt and FL scores were decreased, and the patients’ ocular surface function was significantly improved, which was conducive to recovering the tear film stability. Moreover, the total effective rate and adverse reaction rate were 92.86% and 7.14%, respectively, with high safety. Ling et al.^(27)^ researched the efficacy of rbFGF combined with sodium hyaluronate artificial tears in treating dry eyes after surgery and found that bFGF combined with artificial tears (observation group) displayed better ocular surface function, greater improvement in inflammatory factors, and higher total effective rate of treatment. Hei et al.^(28)^ explored the therapeutic effect of rbFGF eye drops combined with artificial tears on dry eyes. The results manifested that the total effective rate in the observation group (95.12%) treated with rbFGF eye drops combined with artificial tears was significantly higher than that in the control group (82.92%) treated with artificial tears, and the improvement in symptom score and ocular surface function was markedly superior to that in the control group. Consistently, we found that the clinical symptom score decreased significantly in both the groups after treatment when compared with that before treatment, and it was significantly lower in the observation group than in the control group.

Compensatory oxidative stress occurs at the initial stage of cataract, and long-term oxidative stress damage sharply decreases SOD content, which reduces the total antioxidant capacity and increases the proportion of highly active free radicals. Free radicals can interact with polyunsaturated fatty acids in the cell membranes to form LPO, which then decomposes into large amounts of aldehydes, alcohols, and hydrocarbons. The resulting MDA has strong cytotoxicity and forms stable insoluble metabolic end-products with phospholipid proteins and nucleic acids, which continuously accumulate in cells to affect their functions. On the other hand, LPO also elevates the fluidity, fragility, and permeability of biomem-branes, causing cell dysfunction and damage to the basic structure. Similarly, we found that the observation group showed significantly reduced TNF-α, IL-6, MDA, and LPO levels and increased SOD and TAC after treatment, and these inflammatory and oxidative stress indices of the observation group showed significant improvement relative to those of the control group.

Currently, there are only a few studies on the impacts of preoperative intervention in dry eye symptoms on the postoperative ocular surface function and inflammatory factor levels in cataract patients complicated with dry eyes. Following treatment, the clinical symptom score, OSDI, FL score, TNF-*, IL-6, MDA, and LPO significantly decreased, and BUT, SIt score, SOD, and TAC significantly increased in both the control and observation groups, and the clinical symptom score, as well as the markers of ocular surface function and inflammatory factors, improved more rapidly in the observation group than in the control group.

In conclusion, the artificial tears combined with rbFGF before operation can remarkably ameliorate the ocular surface function, lower the levels of inflammatory factors in tears, and relieve dry eye symptoms after operation in cataract patients complicated with dry eyes. Our findings thus provide a reference for the clinical treatment of such patients.
